# Minimising the Kullback–Leibler Divergence for Model Selection in Distributed Nonlinear Systems

**DOI:** 10.3390/e20020051

**Published:** 2018-01-23

**Authors:** Oliver M. Cliff, Mikhail Prokopenko, Robert Fitch

**Affiliations:** 1 Australian Centre for Field Robotics, The University of Sydney, Sydney NSW 2006, Australia; 2 Complex Systems Research Group, The University of Sydney, Sydney NSW 2006, Australia; 3 Centre for Autonomous Systems, University of Technology Sydney, Ultimo NSW 2007, Australia

**Keywords:** Kullback–Leibler divergence, model selection, information theory, transfer entropy, stochastic interaction, nonlinear systems, complex networks, state space reconstruction

## Abstract

The Kullback–Leibler (KL) divergence is a fundamental measure of information geometry that is used in a variety of contexts in artificial intelligence. We show that, when system dynamics are given by distributed nonlinear systems, this measure can be decomposed as a function of two information-theoretic measures, transfer entropy and stochastic interaction. More specifically, these measures are applicable when selecting a candidate model for a distributed system, where individual subsystems are coupled via latent variables and observed through a filter. We represent this model as a directed acyclic graph (DAG) that characterises the unidirectional coupling between subsystems. Standard approaches to structure learning are not applicable in this framework due to the hidden variables; however, we can exploit the properties of certain dynamical systems to formulate exact methods based on differential topology. We approach the problem by using reconstruction theorems to derive an analytical expression for the KL divergence of a candidate DAG from the observed dataset. Using this result, we present a scoring function based on transfer entropy to be used as a subroutine in a structure learning algorithm. We then demonstrate its use in recovering the structure of coupled Lorenz and Rössler systems.

## 1. Introduction

Distributed information processing systems are commonly studied in complex systems and machine learning research. We are interested in inferring data-driven models of such systems, specifically in the case where each subsystem can be viewed as a nonlinear dynamical system. In this context, the Kullback–Leibler (KL) divergence is commonly used to measure the quality of a statistical model [1,2,3]. When a model is compared with fully observed data, computing the KL divergence can be straightforward. However, in the case of spatially distributed dynamical systems, where individual subsystems are coupled via latent variables and observed through a filter, the presence of hidden variables renders typical approaches unusable. We derive the KL divergence in such systems as a function of two information-theoretic measures using methods from differential topology.

The *model selection* problem has applications in a wide variety of areas due to its usefulness in performing efficient inference and understanding the underlying phenomena being studied. Dynamical systems are an expressive model characterised by a map that describes their evolution over time and a read-out function through which we observe the latent state. Our research focuses on the more general case of a multivariate system, where a set of these subsystems are distributed and unidirectionally coupled to one another. The problem of inferring this coupling is an important multidisciplinary study in fields such as ecology [4], neuroscience [5,6], multi-agent systems [7,8,9], and various others that focus on artificial and biological networks [10].

We represent such a spatially distributed system as a probabilistic graphical model termed a *synchronous graph dynamical system (GDS)* [11,12], whose structure is given by a DAG. Model selection in this context is the problem of inferring directed relationships between hidden variables from an observed dataset, also known as *structure learning*. A main challenge in structure learning for DAGs is the case where variables are unobserved. Exact methods are known for fully observable systems (i.e., Bayesian network (BNs)) [13]; however, these are not applicable in the more expressive case when the state variables in dynamical systems are latent. The main focus of this paper is to analytically derive a measure for comparing a candidate graph to the underlying graph that generated a measured dataset. Such a measure can then be used to solve the two subproblems that comprise structure learning, *evaluation* and *identification* [14], and hence find the optimal model that explains the data.

For the evaluation problem, it is desirable to select the *simplest* model that incorporates all statistical knowledge. This concept is commonly expressed via information theory, where an established technique is to evaluate the encoding length of the data, given the model [1,15,16]. The simplest model should aim to minimise code length [2], and therefore we can simplify our problem to that of minimising KL divergence for the synchronous GDS. Using this measure, we find a factorised distribution (given by the graph structure) that is closest to the complete (unfactorised) distribution. We first analytically derive an expression for this divergence, and build on this result to present a scoring function for evaluating candidate graphs based on a dataset.

**Figure 1:**
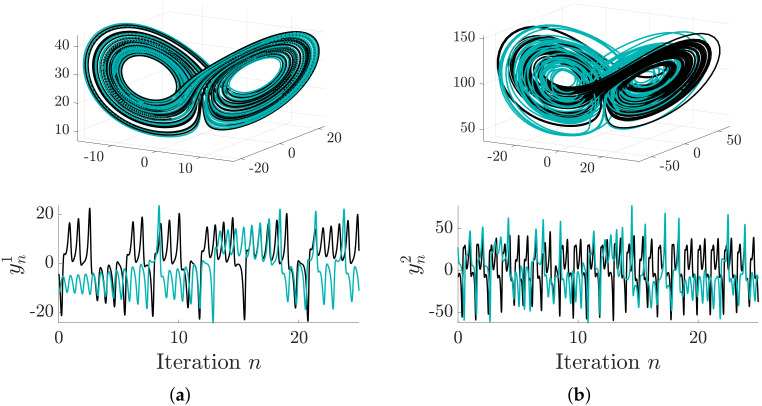
Trajectory of a pair of coupled Lorenz systems. *Top row*: original state of the subsystems. *Bottom row:* time-series measurements of the subsystems. In each figure, the black lines represent an uncoupled simulation (λ=0), and teal lines illustrate a simulation where the first (leftmost) subsystem was coupled to the second (λ=10). (**a**) σ=10,β=8/3,ρ=28; (**b**) σ=10,β=8/3,ρ=90.

The main result of this paper is an exact decomposition of the KL divergence for synchronous GDSs. We show that this measure can be decomposed as the difference between two well-known information-theoretic measures, stochastic interaction [17,18] and collective transfer entropy [19]. We establish this result by first representing discrete-time multivariate dynamical systems as dynamic Bayesian network (DBNs) [20]. In this form, both the complete and factorised distributions cannot be directly computed due to the hidden system state. Thus, we draw on state space reconstruction methods from differential topology to reformulate the KL divergence in terms of computable distributions. Using this expression, we show that the maximum transfer entropy graph is the most likely to have generated the data. This is experimentally validated using toy examples of a Lorenz–Rössler system and a network of coupled Lorenz attractors (Figure 1) of up to four nodes. These results support the conjecture that transfer entropy can be used to infer effective connectivity in complex networks.

## 2. Related Work

Networks of coupled dynamical systems have been introduced under a variety of terms, such as complex networks [10], distributed dynamical systems [6] and master–slave configurations [21]. The defining feature of these networks is that the dynamics of each subsystem are given by a set of either discrete-time maps or first-order ordinary differential equation (ODEs). In this paper, we use the discrete-time formulation, where a map can be obtained numerically by integrating ODEs or recording observations at discrete-time intervals [22].

An important precursor to network reconstruction is inferring causality and coupling strength between complex nonlinear systems. Causal inference is intractable when the experimenter can not intervene with the dataset [23], and so we focus our attention on methods that determine conditional independence (coupling) rather than causality. In seminal work, Granger [24] proposed *Granger causality* for quantifying the predictability of one variable from another; however, a key requirement of this measure is linearity of the system, implying subsystems are separable [4]. Schreiber [25] extended these ideas and introduced *transfer entropy* using the concept of finite-order Markov processes to quantify the information transfer between coupled nonlinear systems. Transfer entropy and Granger causality are equivalent for linearly-coupled Gaussian systems (e.g., Kalman models) [26]; however, there are clear distinctions between the concepts of information transfer and causal effect [27]. Although transfer entropy has received criticism over spuriously identifying causality [28,29,30], we are concerned with statistical modelling and not causality of the underlying process.

Recently, a number of measures have been proposed to infer coupling between distributed dynamical systems based on reconstruction theorems. Sugihara et al. [4] proposed convergent cross-mapping that involves collecting a history of observed data from one subsystem and uses this to predict the outcome of another subsystem. This history is the delay reconstruction map described by Takens’ Delay Embedding Theorem [31]. Similarly, Schumacher et al. [6] used the Bundle Delay Embedding Theorem [32,33] to infer causality and perform inference via Gaussian processes. Although the algorithms presented in these papers can infer driving subsystems in a spatially distributed dynamical system, the results obtained differ from ours as inference is not considered for an entire network structure, nor is a formal derivation presented. Contrasting this, we recently derived an information criterion for learning the structure of distributed dynamical systems [12]. However, the criterion proposed required parametric modelling of the probability distributions, and thus a detailed understanding of the physical phenomena being studied. In this paper, we extend this framework by first showing that KL divergence can be decomposed as information-theoretically useful measures, and then arriving at a similar result but employing non-parametric density estimation techniques to allow for no assumptions about the underlying distributions.

It is important to distinguish our approach from dynamic causal modelling (DCM), which attempts to infer the parameters of explicit dynamic models that cause (generate) data. In DCM, the set of potential models is specified a priori (typically in the form of ODEs) and then scored via marginal likelihood or evidence. The parameters of these models include *effective connectivity* such that their posterior estimates can be used to infer coupling among distributed dynamical systems [34]. As a consequence, these approaches can be used to recover networks that reveal the effective structure of observed systems [35,36]. In contrast, our approach does not require an explicitly specified model because the scoring function can be computed directly from the data. However, it does assume an implicit model in the form of a DAG where the subsystem processes are generated by generic functions.

Unlike effective connectivity, which is defined in relation to a (dynamic causal) model, the concept of *functional connectivity* refers to recovering statistical dependencies [37]. Consequently, statistical measures such as Granger causality and transfer entropy are typically used to identify functional, rather than effective structure. For example, transfer entropy has been used previously to infer networks in numerous fields, e.g., computational neuroscience [5,38], multi-agent systems [8], financial markets [39], supply-chain networks [40], and biology [41]. However, most of these results build on the work of Schreiber [25] by assuming the system is composed of finite-order Markov chains and thus there is a dearth of work that provides formal derivations for the use of this measure for inferring effective connectivity. Our work allows us to compute scoring functions directly from multivariate time series (as in functional connectivity), yet still assumes an implicit model (albeit with weaker assumptions on the model than those considered in inferring effective connectivity).

## 3. Background

### 3.1. Notation

We use the convention that (·) denotes a sequence, {·} a set, and 〈·〉 a vector. In this work, we consider a collection of stationary stochastic temporal processes Z. Each process $Z^{i}$ comprises a sequence of random variables $\left(Z_{1}^{i},\ldots ,Z_{N}^{i}\right)$ with realisation $\left(z_{1}^{i},\ldots ,z_{N}^{i}\right)$ for countable time indices n∈N. Given these processes, we can compute probability distributions of each variable by counting relative frequencies or by density estimation techniques [42,43]. We use bold to denote the set of all variables, e.g., $z_{n}=\left\{z_{n}^{1},\ldots ,z_{n}^{M}\right\}$ is the collection of *M* realisations at index *n*. Furthermore, unless otherwise stated, $X_{n}^{i}$ is a latent (hidden) variable, $Y_{n}^{i}$ is an observed variable, and $Z_{n}^{i}$ is an arbitrary variable; thus, $Z_{n}=\left\{X_{n},Y_{n}\right\}$ is the set of all hidden and observed variables at temporal index *n*. Given a graphical model *G*, the $p^{i}$ parents of variable $Z_{n+1}^{i}$ are given by the parent set $\Pi_{G}\left(Z_{n+1}^{i}\right)={\left\{Z_{n}^{ij}\right\}}_{j}=\left\{Z_{n}^{i1},\ldots ,Z_{n}^{ip^{i}}\right\}$. Finally, let the superscript $z_{n}^{i,(k)}=\langle z_{n}^{i},z_{n-1}^{i},\ldots ,z_{n-k+1}^{i}\rangle$ denote the vector of *k* previous values taken by variable $Z_{n}^{i}$.

### 3.2. Representing Distributed Dynamical Systems as Probabilistic Graphical Models

**Figure 2:**
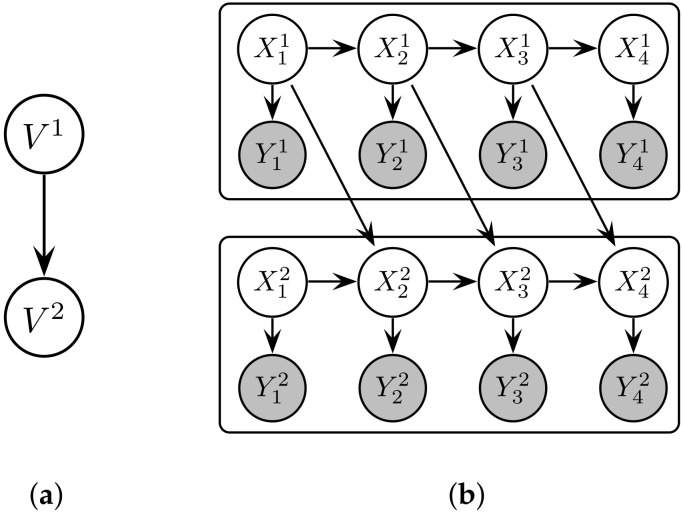
Representation of (**a**) the synchronous GDS with two vertices ($V^{1}$ and $V^{2}$), and (**b**) the rolled-out DBN of the equivalent structure. Subsystems $V^{1}$ and $V^{2}$ are coupled by virtue of the edge $X_{n}^{1}\to X_{n+1}^{2}$.

We are interested in modelling discrete-time multivariate dynamical systems, where the state is a vector of real numbers given by a point $x_{n}$ lying on a compact *d*-dimensional manifold M. A map f:M→M describes the temporal evolution of the state at any given time, such that the state at the next time index $x_{n+1}=f\left(x_{n}\right)$. Furthermore, in many practical scenarios, we do not have access to $x_{n}$ directly, and can instead observe it through a *measurement function* $\psi :M\to R^{M}$ that yields a scalar representation $y_{n}=\psi \left(x_{n}\right)$ of the latent state [22,44]. We assume the multivariate system can be factorised and modelled as a DAG with spatially distributed dynamical subsystems, termed a synchronous GDS (see Figure 2a). This definition is restated from [12] as follows.

**Definition** **1.** (Synchronous GDS) *A synchronous GDS $\left(G,x_{n},y_{n},\left\{f^{i}\right\},\left\{\psi^{i}\right\}\right)$ is a tuple that consists of: a finite, directed graph G=(V,E) with edge-set $E=\{E^{i}\}$ and M vertices comprising the vertex set $V=\{V^{i}\}$; a multivariate state $x_{n}=\langle x_{n}^{i}\rangle$, composed of states for each vertex $V^{i}$ confined to a $d^{i}$-dimensional manifold $x_{n}^{i}\in M^{i}$; an M-variate observation $y_{n}=\langle y_{n}^{i}\rangle$, composed of scalar observations for each vertex $y_{n}^{i}\in R$; a set of local maps $\left\{f^{i}\right\}$ of the form $f^{i}:M\to M^{i}$, which update synchronously and induce a global map f:M→M; and a set of local observation functions $\left\{\psi^{1},\psi^{2},\ldots ,\psi^{M}\right\}$ of the form $\psi^{i}:M^{i}\to R$.*

The global dynamics and observations can therefore be described by the set of local functions [12]:

(1) $$\begin{array}{c} x_{n+1}^{i}=f^{i}\left(x_{n}^{i},{\langle x_{n}^{ij}\rangle }_{j}\right)+\upsilon_{f^{i}}, \end{array}$$

(2) $$\begin{array}{c} y_{n+1}^{i}=\psi^{i}\left(x_{n+1}^{i}\right)+\upsilon_{\psi^{i}}, \end{array}$$

where $\upsilon_{f^{i}}$ and $\upsilon_{\psi^{i}}$ are additive noise terms. The subsystem dynamics (1) are a function of the subsystem state $x_{n}^{i}$ and the subsystem parents’ state ${\langle x_{n}^{ij}\rangle }_{j}$ at the previous time index, i.e., $f^{i}:\left(M^{i}\times_{j}M^{ij}\right)\to M^{i}$. However, the observation $y_{n+1}^{i}$ is a function of the subsystem state alone, i.e., $\psi^{i}:M^{i}\to R$. We assume that the maps $\left\{f^{i}\right\}$ and $\left\{\psi^{i}\right\}$, as well as the graph *G*, are time-invariant.

The discrete-time mapping for the dynamics (1) and measurement functions (2) can be modelled as a DBN in order to facilitate structure learning of the graph [12] (see Figure 2b). DBNs are a probabilistic graphical model that represent probability distributions over trajectories of random variables $\left(Z_{1},Z_{2},\ldots \right)$ using a prior BN and a *two-time-slice BN (2TBN)* [45]. To model the maps, however, we need only to consider the 2TBN $B=(G,\Theta_{G})$, which can model a first-order Markov process $p_{B}\left(z_{n+1}|z_{n}\right)$ graphically via a DAG *G* and a set of conditional probability distribution (CPD) parameters $\Theta_{G}$ [45]. Given a set of stochastic processes $\left(Z_{1},Z_{2},\ldots ,Z_{N}\right)$, the realisation of which constitutes the sample path $\left(z_{1},z_{2},\ldots ,z_{N}\right)$, the 2TBN distribution is given by $p_{B}\left(z_{n+1}|z_{n}\right)=\prod_{i}\mathrm{Pr}\left(z_{n+1}^{i}|\pi_{G}\left(Z_{n+1}^{i}\right)\right),$ where $\pi_{G}\left(Z_{n+1}^{i}\right)$ denotes the (index-ordered) set of realisations $\left\{z_{o}^{j}:Z_{o}^{j}\in \Pi_{G}\left(Z_{n+1}^{i}\right)\right\}$.

To model the synchronous GDS as a DBN, we associate each subsystem vertex $V^{i}$ with a state variable $X_{n}^{i}$ and an observation variable $Y_{n}^{i}$. The parents of subsystem $V^{i}$ are denoted $\Pi_{G}\left(V^{i}\right)$ [12]. From the dynamics (1), variables in the set $\Pi_{G}\left(X_{n+1}^{i}\right)$ come strictly from the preceding time slice, and additionally, from the measurement function (2), $\Pi_{G}\left(Y_{n+1}^{i}\right)=X_{n+1}^{i}$. Thus, we can build the edge set E in the GDS by means of the edges in the DBN [12], i.e., given an edge $X_{n}^{i}\to X_{n+1}^{j}$ of the DBN, the equivalent edge $V^{i}\to V^{j}$ exists for the GDS. The distributions for the dynamics (1) and observation (2) maps of *M* arbitrary subsystems can therefore be factorised according to the DBN structure such that [12]

(3) $$p_{B}\left(z_{n+1}|z_{n}\right)=\prod_{i=1}^{M}\mathrm{Pr}\left(x_{n+1}^{i}|x_{n}^{i},{\langle x_{n}^{ij}\rangle }_{j}\right)\cdot \mathrm{Pr}\left(y_{n+1}^{i}|x_{n+1}^{i}\right).$$

The goal of learning nonlinear dynamical networks thus becomes that of inferring the parent set $\Pi_{G}\left(X_{n}^{i}\right)$ for each latent variable $X_{n}^{i}$.

Finally, recall that the parents of each observation are constrained such that $\Pi_{G}\left(Y_{n+1}^{i}\right)=X_{n+1}^{i}$. As a consequence, we use the shorthand notation $y_{n}^{ij}$ to denote the observation of the *j*-th parent of the *i*-th subsystem at time *n* (and the same for $x_{n}^{ij}$).

### 3.3. Network Scoring Functions

A number of exact and approximate DBN structure learning algorithms exist that are based on Bayesian statistics and information theory. We have shown in prior work how to compute the log-likelihood function for synchronous GDSs. In this section, we will briefly summarise the problem of structure learning for DBNs, focusing on the factorised distribution (3).

The *score and search* paradigm [46] is a common method for recovering graphical models from data. Given a dataset $D=(y_{1},y_{2},\ldots ,y_{N})$, the objective is to find a DAG $G^{*}$ such that

(4) $$G^{*}=\underset{G\in G}{arg max}g\left(B:D\right),$$

where g(B:D) is a scoring function measuring the degree of fitness of a candidate DAG *G* to the data set *D*, and G is the set of all DAGs. Finding the optimal graph $G^{*}$ in Equation (4) requires solutions to the two subproblems that comprise structure learning: the *evaluation* problem and the *identification* problem [14]. The main problem we focus on in this paper is the evaluation problem, i.e., determining a score that quantifies the quality of a graph, given data. Later, we will address the identification problem by discussing the attributes of this scoring function in efficiently finding the optimal graph structure.

In prior work, we developed a score based on the posterior probability of the network structure *G*, given data *D*. That is, we considered maximising the expected log-likelihood [12]

(5) $$\ell \left({\hat{\Theta }}_{G}:D\right)=E\;[\log\mathrm{Pr}(D|G,{\hat{\Theta }}_{G})]=E\;[\log(p_{B}\left(z_{n+1}|z_{n}\right))],$$

where the expectation $E\left[Z\right]=\int_{-\infty }^{\infty }z\mathrm{Pr}\left(z\right)dz$. It was shown that state space reconstruction techniques (see Appendix A) can be used to compute the log-likelihood of Equation (3) as a difference of conditional entropy terms [12]. In the same work, we illustrated that the log-likelihood ratio of a candidate DAG *G* to the empty network $G_{\emptyset }$ is given by collective transfer entropy (see Appendix B), i.e.,

(6) $$\ell \left({\hat{\Theta }}_{G}:D\right)-\ell \left({\hat{\Theta }}_{G_{\emptyset }}:D\right)=N\cdot \sum_{i=1}^{M}T_{{\langle Y^{ij}\rangle }_{j}\to Y^{i}}.$$

For the nested log-likelihoods above, the statistics of $2(\ell \left({\hat{\Theta }}_{G}:D\right)-\ell \left({\hat{\Theta }}_{G_{\emptyset }}:D\right))$ asymptotically follow the $\chi_{q}^{2}$-distribution, where *q* is the difference between the number of parameters of each model [47,48]. We will draw on this log-likelihood decomposition in later sections for statistical significance testing.

## 4. Computing Conditional KL Divergence

In this section, we present our main result, which is an analytical expression of KL divergence that facilitates structure learning in distributed nonlinear systems. We begin by considering the problem of finding an optimal DBN structure as searching for a parsimonious *factorised distribution* $p_{B}$ that best represents the complete digraph distribution $p_{K_{M}}$. That is, $p_{K_{M}}$ is the joint distribution yielded by assuming no factorisation (the complete graph $K_{M}$) and thus no information loss. The distribution is expressed as:

(7) $$p_{K_{M}}\left(z_{n+1}|z_{n}^{\left(n\right)}\right)=\mathrm{Pr}(\left\{z_{n+1}^{1},\ldots ,z_{n+1}^{M}\right\}|\left\{z_{n}^{1},\ldots ,z_{n}^{M}\right\},\left\{z_{n-1}^{1},\ldots ,z_{n-1}^{M}\right\},\left\{z_{1}^{1},\ldots ,z_{1}^{M}\right\})\;.$$

We quantify the similarity of the factorised distribution $p_{B}$ to this joint distribution via KL divergence. In prior work, De Campos [3] derived the *MIT* scoring function for BNs by this approach and it was later used for DBN structure learning with complete data [49]. We extend the analysis to DBNs with latent variables, i.e., we compare the joint and factorised distributions of time slices, given the entire history,

(8) $$\begin{array}{ll} D_{\mathrm{KL}}\left[p_{K_{M}}∥p_{B}\right] & =D_{\mathrm{KL}}\left[p_{K_{M}}\left(z_{n+1}|z_{n}^{\left(n\right)}\right)∥p_{B}\left(z_{n+1}|z_{n}^{\left(n\right)}\right)\right]\\ & =\sum_{z_{n}^{\left(n\right)}}\mathrm{Pr}\left(z_{n}^{\left(n\right)}\right)\sum_{z_{n+1}}\mathrm{Pr}\left(z_{n+1}|z_{n}^{\left(n\right)}\right)\log\frac{\mathrm{Pr}(z_{n+1}|z_{n}^{\left(n\right)})}{p_{B}\left(z_{n+1}|z_{n}^{\left(n\right)}\right)}\\ & =E\left[\log\frac{\mathrm{Pr}(z_{n+1}|z_{n}^{\left(n\right)})}{p_{B}\left(z_{n+1}|z_{n}\right)}\right]. \end{array}$$

Substituting the synchronous GDS model (3) into Equation (8), we get

(9) $$D_{\mathrm{KL}}\left[p_{K_{M}}∥p_{B}\right]=E\left[\log\frac{\mathrm{Pr}(z_{n+1}|z_{n}^{\left(n\right)})}{\prod_{i=1}^{M}\mathrm{Pr}\left(x_{n+1}^{i}|x_{n}^{i},{\langle x_{n}^{ij}\rangle }_{j}\right)\cdot \mathrm{Pr}\left(y_{n+1}^{i}|x_{n+1}^{i}\right)}\right].$$

However, Equation (9) comprises maximum likelihood distributions with unobserved (latent) states $x_{n}$. It is common in model selection to decompose the KL divergence as

(10) $$D_{\mathrm{KL}}\left[p_{K_{M}}∥p_{B}\right]=E\left[\log\left(\mathrm{Pr}(z_{n+1}|z_{n}^{\left(n\right)})\right)\right]-[\log(p_{B}\left(z_{n+1}|z_{n}\right))],$$

where the second term is simply the log-likelihood (5). In this form, $p_{K_{M}}$ is often identical for all models considered and, in practice, it suffices to ignore this term and thus avoid the problem of computing distributions of latent variables. The resulting simpler expression can be viewed as log-likelihood maximisation (as in our previous work outlined in Section 3.3). However, as we show in this section, $p_{K_{M}}$ is not equivalent for all models unless certain parameters of the dynamical systems are known. Hence, for now, we cannot ignore the first term of Equation (10) and we instead propose an alternative decomposition of KL divergence that comprises only observed variables.

### 4.1. A Tractable Expression via Embedding Theory

In order to compute the distributions in (9), we use the Bundle Delay Embedding Theorem [32,33] to reformulate the factorised distribution (denominator), and the Delay Embedding Theorem for Multivariate Observation Functions [50] for the joint distribution (numerator). We describe these theorems in detail in Appendix A, along with the technical assumptions required for (f,ψ). Although the following theorems assume a diffeomorphism, we also discuss application of the theory towards inferring the structure of endomorphisms (e.g., coupled map lattices [51]) in the same appendix.

The first step is to reproduce a prior result for computing the factorised distribution (denominator) in Equation (9). First, the embedding

(11) $$y_{n}^{i,(\kappa^{i})}=\langle y_{n}^{i},y_{n-\tau^{i}}^{i},\ldots ,y_{n-\left(\kappa^{i}-1\right)\tau^{i}}^{i}\rangle ,$$

where $\tau^{i}$ is the (strictly positive) lag, and $\kappa^{i}$ is the embedding dimension of the *i*-th subsystem (the *embedding parameters*). Note that, although we can take either the future or past delay embedding (11) for diffeomorphisms, we explicitly consider a *history* of values to account for both endomorphisms and diffeomorphisms. Moreover, an important assumption of our approach is that the the structure (enforced by coupling between subsystems) is a DAG; this comes from the Bundle Delay Embedding Theorem [32,33] (see Lemma 1 of [12] for more detail). Our previous result is expressed as follows.

**Lemma** **1** (Cliff et al. [12]). *Given an observed dataset D, where $y_{n}\in R^{M}$, generated by a directed and acyclic synchronous GDS $\left(G,x_{n},y_{n},\left\{f^{i}\right\},\left\{\psi^{i}\right\}\right)$, the 2TBN distribution can be written as*

(12) $$\prod_{i=1}^{M}\mathrm{Pr}\left(x_{n+1}^{i}|x_{n}^{i},{\langle x_{n}^{ij}\rangle }_{j}\right)\cdot \mathrm{Pr}\left(y_{n+1}^{i}|x_{n+1}^{i}\right)=\frac{\prod_{i=1}^{M}\mathrm{Pr}\left(y_{n+1}^{i}|y_{n}^{i,(\kappa^{i})},{\langle y_{n}^{ij,(\kappa^{ij})}\rangle }_{j}\right)}{\mathrm{Pr}(x_{n}|\langle y_{n}^{i,(\kappa^{i})}\rangle )}.$$

Next, we present a method for computing the joint distribution (numerator) in Lemma 3. For convenience, Lemma 2 restates part of the delay embedding theorem in [50] in terms of subsystems of a synchronous GDS and establishes existence of a map G for predicting future observations from a history of observations.

**Lemma** **2.** Consider a diffeomorphism f:M→M on a d-dimensional manifold M, where the multivariate state $x_{n}$ consists of M subsystem states $\langle x_{n}^{1},\ldots ,x_{n}^{M}\rangle$. Each subsystem state $x_{n}^{i}$ is confined to a submanifold $M^{i}\subseteq M$ of dimension $d^{i}\leq d$, where $\sum_{i}d^{i}=d$. The multivariate observation is given, for some map G, by $y_{n+1}=G\left(\langle y_{n}^{i,(\kappa^{i})}\rangle \right)$.

**Proof.**  The proof restates part of the proof of Theorem 2 of Deyle and Sugihara [50] in terms of subsystems.

Given *M* inhomogeneous observation functions $\left\{\psi^{i}\right\}$, the following map

(13) $$\Phi_{f,\psi }\left(x\right)=\langle \Phi_{f^{1},\psi^{1}}\left(x\right),\Phi_{f^{2},\psi^{2}}\left(x\right),\ldots ,\Phi_{f^{M},\psi^{M}}\left(x\right)\rangle$$

is an embedding where each subsystem (local) map $\Phi_{f^{i},\psi^{i}}:M\to R^{\kappa^{i}}$, smoothly (at least $C^{2}$), and, at time index *n* is described by

(14) $$\begin{array}{cc} \Phi_{f^{i},\psi^{i}}\left(x_{n}\right) & =\langle \psi^{i}\left(x_{n}\right),\psi^{i}\left(x_{n-\tau }\right),\ldots ,\psi^{i}\left(x_{n-(k-1)\tau }\right)\rangle \\ & =y_{n}^{i,(\kappa^{i})}, \end{array}$$

where $\sum_{i}\kappa^{i}=2d+1$ [50]. Note that, from (13) and (14), we have the global map

$$\Phi_{f,\psi }\left(x_{n}\right)=\langle y_{n}^{i,(\kappa^{i})}\rangle =\langle y_{n}^{1,(\kappa^{1})},\ldots ,y_{n}^{m,(\kappa^{M})}\rangle .$$

Now, since $\Phi_{f,\psi }$ is an embedding, it follows that the map $F=\Phi_{f,\psi }\circ f\circ \Phi_{f,\psi }^{-1}$ is well defined and a diffeomorphism between two observation sequences $F:R^{2d+1}\to R^{2d+1}$, i.e.,

$$\begin{array}{cc} \langle y_{n+1}^{i,(\kappa^{i})}\rangle & =\Phi_{f,\psi }\left(x_{n+1}\right)=\Phi_{f,\psi }\left(f\left(x_{n}\right)\right)\\ & =\Phi_{f,\psi }\left(f\left(\Phi_{f,\psi }^{-1}\left(\langle y_{n}^{i,(\kappa^{i})}\rangle \right)\right)\right)=F\left(\langle y_{n}^{i,(\kappa^{i})}\rangle \right). \end{array}$$

The last 2d+1 components of F are trivial, i.e., the set $\langle y_{n}^{i,(\kappa^{i})}\rangle$ is observed; denote the first *M* components by $G:\Phi_{f,\psi }\to R^{M}$, and then we have $y_{n+1}=G\left(\langle y_{n}^{i,(\kappa^{i})}\rangle \right)$. ☐

We now use the result of Lemma 2 to obtain a computable form of the KL divergence.

**Lemma** **3.** *Consider a discrete-time multivariate dynamical system with generic (f,ψ) modelled as a directed and acyclic synchronous GDS $\left(G,x_{n},y_{n},\left\{f^{i}\right\},\left\{\psi^{i}\right\}\right)$ with M subsystems. The KL divergence of a candidate graph G from the observed dataset D can be computed from tractable probability distributions:*

(15) $$D_{\mathrm{KL}}\left[p_{K_{M}}∥p_{B}\right]=E\left[\log\frac{\mathrm{Pr}(y_{n+1}|\langle y_{n}^{i,(\kappa^{i})}\rangle )}{\prod_{i=1}^{M}\mathrm{Pr}\left(y_{n+1}^{i}|y_{n}^{i,(\kappa^{i})},{\langle y_{n}^{ij,(\kappa^{ij})}\rangle }_{j}\right)}\right].$$

**Proof.**  Lemma 1, we can substitute (12) into (9), and express the KL divergence $D_{\mathrm{KL}}\left[p_{K_{M}}∥p_{B}\right]$ as

(16) $$D_{\mathrm{KL}}\left[p_{K_{M}}∥p_{B}\right]=E\left[\log\left(\mathrm{Pr}\left(z_{n+1}|z_{n}^{\left(n\right)}\right)\cdot \frac{\mathrm{Pr}(x_{n}|\langle y_{n}^{i,(\kappa^{i})}\rangle )}{\prod_{i=1}^{M}\mathrm{Pr}\left(y_{n+1}^{i}|y_{n}^{i,(\kappa^{i})},{\langle y_{n}^{ij,(\kappa^{ij})}\rangle }_{j}\right)}\right)\right].$$

We now focus on $p_{K_{M}}\left(z_{n+1}|z_{n}^{\left(n\right)}\right)$. Using the chain rule,

$$p_{K_{M}}\left(z_{n+1}|z_{n}^{\left(n\right)}\right)=\mathrm{Pr}\left(x_{n+1}|z_{n}^{\left(n\right)}\right)\cdot \mathrm{Pr}\left(y_{n+1}|x_{n+1},z_{n}^{\left(n\right)}\right).$$

Given the Markov property of the dynamics (1) and observation (2) maps, we get

(17) $$p_{K_{M}}\left(z_{n+1}|z_{n}^{\left(n\right)}\right)=\mathrm{Pr}\left(X_{n+1}=f\left(x_{n}\right)|x_{n}\right)\cdot \mathrm{Pr}\left(Y_{n+1}=\psi \left(x_{n+1}\right)|x_{n+1}\right).$$

Now, recall fom Lemma 2 that global equations for the entire system state $x_{n}$ and observation $y_{n}$ are

(18) $$\begin{array}{cc} x_{n+1} & =f\left(x_{n}\right)+\upsilon_{f}=f\left(\Phi_{f,\psi }^{-1}\left(\langle y_{n}^{i,(\kappa^{i})}\rangle \right)\right)+\upsilon_{f}, \end{array}$$

(19) $$\begin{array}{cc} y_{n+1} & =\psi \left(x_{n+1}\right)+\upsilon_{\psi }=G\left(\langle y_{n}^{i,(\kappa^{i})}\rangle \right)+\upsilon_{\psi }. \end{array}$$

Given the assumption of i.i.d noise on the function *f*, from (18), we express the probability of the dynamics $x_{n+1}$, given by the embedding, as

(20) $$\begin{array}{cc} \mathrm{Pr}\left(x_{n+1}|\langle y_{n}^{i,(\kappa^{i})}\rangle \right) & =\mathrm{Pr}\left(X_{n+1}=f\left(\Phi_{f,\psi }^{-1}\left(\langle y_{n}^{i,(\kappa^{i})}\rangle \right)\right)|\langle y_{n}^{i,(\kappa^{i})}\rangle \right)\\ & =\mathrm{Pr}\left(X_{n}=\Phi_{f,\psi }^{-1}\left(\langle y_{n}^{i,(\kappa^{i})}\rangle \right)|\langle y_{n}^{i,(\kappa^{i})}\rangle \right)\cdot \mathrm{Pr}\left(X_{n+1}=f\left(x_{n}\right)|x_{n}\right). \end{array}$$

By assumption, the observation noise is i.i.d or dependent only on the state $x_{n+1}$, and thus the probability of observing $y_{n+1}$, from (19) is

(21) $$\begin{array}{cc} \mathrm{Pr}\left(y_{n+1}|\langle y_{n}^{i,(\kappa^{i})}\rangle \right) & =\mathrm{Pr}\left(Y_{n+1}=G\left(\langle y_{n}^{i,(\kappa^{i})}\rangle \right)|\langle y_{n}^{i,(\kappa^{i})}\rangle \right)\\ & =\mathrm{Pr}\left(X_{n+1}=f\left(\Phi_{f,\psi }^{-1}\left(\langle y_{n}^{i,(\kappa^{i})}\rangle \right)\right)|\langle y_{n}^{i,(\kappa^{i})}\rangle \right)\\ & \;\times \mathrm{Pr}\left(Y_{n+1}=\psi \left(x_{n+1}\right)|x_{n+1}\right). \end{array}$$

By (20) and (21), we have that

(22) $$\mathrm{Pr}\left(x_{n+1}|x_{n}\right)\cdot \mathrm{Pr}\left(y_{n+1}|x_{n+1}\right)=\frac{\mathrm{Pr}(y_{n+1}|\langle y_{n}^{i,(\kappa^{i})}\rangle )}{\mathrm{Pr}(x_{n}|\langle y_{n}^{i,(\kappa^{i})}\rangle )}.$$

Substituting Equation (22) into (17) gives

(23) $$p_{K_{M}}\left(z_{n+1}|z_{n}^{\left(n\right)}\right)=\frac{\mathrm{Pr}(y_{n+1}|\langle y_{n}^{i,(\kappa^{i})}\rangle )}{\mathrm{Pr}(x_{n}|\langle y_{n}^{i,(\kappa^{i})}\rangle )}.$$

Finally, substituting (23) back into (16) yields the statement of the theorem. ☐

Given all variables in (15) are observed, it is now straightforward to compute KL divergence; however, as we will see, it is more convenient to express (15) as a function of known information-theoretic measures.

### 4.2. Information-Theoretic Interpretation

The main theorem of this paper states KL divergence in terms of transfer entropy and stochastic interaction. These information-theoretic concepts are defined in Appendix B for convenience.

**Theorem** **4.** *Consider a discrete-time multivariate dynamical system with generic (f,ψ) represented as a directed and acyclic synchronous GDS $\left(G,x_{n},y_{n},\left\{f^{i}\right\},\left\{\psi^{i}\right\}\right)$ with M subsystems. The KL divergence $D_{\mathrm{KL}}\left[p_{K_{M}}∥p_{B}\right]$ of a candidate graph G from the observed dataset D can be expressed as the difference between stochastic interaction (A9) and collective transfer entropy (A8), i.e.,*

(24) $$D_{\mathrm{KL}}\left[p_{K_{M}}∥p_{B}\right]=S_{Y}-\sum_{i=1}^{M}T_{{\left\{Y^{ij}\right\}}_{j}\to Y^{i}}.$$

**Proof.**  We can reformulate the KL divergence in (15) as

(25) $$\begin{array}{cc} D_{\mathrm{KL}}\left[p_{K_{M}}∥p_{B}\right] & =E\left[\log\left(\mathrm{Pr}(y_{n+1}|\langle y_{n}^{i,(\kappa^{i})}\rangle )\right)\right]-E\left[\log\left(\prod_{i=1}^{M}\mathrm{Pr}\left(y_{n+1}^{i}|y_{n}^{i,(\kappa^{i})},{\langle y_{n}^{ij,(\kappa^{ij})}\rangle }_{j}\right)\right)\right]\\ & =-H\left(Y_{n+1}|\left\{Y_{n}^{\left(\kappa^{i}\right)}\right\}\right)+\sum_{i=1}^{M}H\left(Y_{n+1}^{i}|Y_{n}^{i,(\kappa^{i})},{\left\{Y_{n}^{ij,(\kappa^{ij})}\right\}}_{j}\right)\\ & =-H\left(Y_{n+1}|\left\{Y_{n}^{\left(\kappa^{i}\right)}\right\}\right)+\sum_{i=1}^{M}H\left(Y_{n+1}^{i}|Y_{n}^{i,(\kappa^{i})}\right)\\ & \;+\sum_{i=1}^{M}\left(H\left(Y_{n+1}^{i}|Y_{n}^{i,(\kappa^{i})},{\left\{Y_{n}^{ij,(\kappa^{ij})}\right\}}_{j}\right)-H\left(Y_{n+1}^{i}|Y_{n}^{i,(\kappa^{i})}\right)\right). \end{array}$$

Substituting in the definitions of transfer entropy (A8) and stochastic interaction (A9) completes the proof. ☐

To conclude this section, we present the following corollary showing that, when we assume a maximum or fixed embedding dimension $\kappa^{i}$ and time delay $\tau^{i}$, it suffices to maximise the collective transfer entropy alone in order to minimise KL divergence for a synchronous GDS.

**Corollary** **1.** *Fix an embedding dimension $\kappa^{i}$ and time delay $\tau^{i}$ for each subsystem $V^{i}\in V$. Then, the graph G that minimises the KL divergence $D_{\mathrm{KL}}\left[p_{K_{M}}∥p_{B}\right]$ is equivalent to the graph that maximises transfer entropy, i.e.,*

(26) $$\underset{G\in G}{arg min}D_{\mathrm{KL}}\left[p_{K_{M}}∥p_{B}\right]=\underset{G\in G}{arg max}\sum_{i=1}^{M}T_{{\left\{Y^{ij}\right\}}_{j}\to Y^{i}}.$$

**Proof.**  The first term of (24) is constant, given a constant vertex set V, time delay τ and embedding dimension κ and is thus unaffected by the parent set $\Pi_{G}\left(V^{i}\right)$ of a variable. As a result, $S_{Y}$ does not depend on the graph *G* being considered, and, therefore, we only need to consider transfer entropy when optimising KL divergence (24). ☐

As mentioned above, Corollary 1 is, in practice, equivalent to the maximum log-likelihood (5) and log-likelihood ratio (6) approaches. However, the statement only holds for constant embedding parameters. In the general case, where these parameters are unknown, one requires Theorem 4 to perform structure learning. Given this result, we can now confidently derive scoring functions from Corollary 1.

## 5. Application to Structure Learning

We now employ the results above in selecting a synchronous GDS that best fits data generated by a multivariate dynamical system. The most natural way to find an optimal model based on Theorem 4 is to minimise KL divergence. Here, we assume constant embedding parameters and use Corollary 1 to present the *transfer entropy score* and discuss some attributes of this score. We then use this scoring function as a subroutine for learning the structure of coupled Lorenz and Rössler attractors.

From Corollary 1, a naive scoring function can be defined as

(27) $$g_{TE}\left(B:D\right)=\sum_{i=1}^{M}T_{{\left\{Y^{ij}\right\}}_{j}\to Y^{i}}.$$

Given parameterised probability distributions, this score is insufficient, since the sum of transfer entropy in (27) is non-decreasing when including more parents in the graph [38]. Thus, we use statistical significance tests in our scoring functions to mitigate this issue.

### 5.1. Penalising Transfer Entropy by Independence Tests

Building on the maximum likelihood score (27), we propose using independence tests to define two new scores of practical value. Here, we draw on the result of de Campos [3], who derived a scoring function for BN structure learning based on conditional mutual information and statistical significance tests, called *MIT*. The central idea is to use collective transfer entropy $T_{{\langle Y^{ij}\rangle }_{j}\to Y^{i}}$ to measure the degree of interaction between each subsystem $V^{i}$ and its parent subsystems $\Pi_{G}\left(V^{i}\right)$, but also to penalise this term with a value based on significance testing. As with the *MIT* score, this gives a principled way to re-scale the transfer entropy when including more edges in the graph.

To develop our scores, we form a *null hypothesis* $H_{0}$ that there is no interaction $T_{{\langle Y^{ij}\rangle }_{j}\to Y^{i}}$, and then compute a test statistic to penalise the measured transfer entropy. To compute the test statistic, it is necessary to consider the measurement distribution in the case where the hypothesis is true. Unfortunately, this distribution is only analytically tractable in the case of discrete and linear-Gaussian systems, where $2NT_{{\langle Y^{ij}\rangle }_{j}\to Y^{i}}$ is known to asymptotically approach the $\chi^{2}$-distribution [48]. Since this distribution is a function of the parents of $Y^{i}$, we let it be described by the function $\chi^{2}\left({\left\{l^{ij}\right\}}_{j}\right)$. Now, given this distribution, we can fix some *confidence level* α and determine the value $\chi_{\alpha ,{\left\{l^{ij}\right\}}_{j}}$ such that $p(\chi^{2}\left({\left\{l^{ij}\right\}}_{j}\right)\leq \chi_{\alpha ,{\left\{l^{ij}\right\}}_{j}})$. This represents a conditional independence test: if $2NT_{{\langle Y^{ij}\rangle }_{j}\to Y^{i}}\leq \chi_{\alpha ,{\left\{l^{ij}\right\}}_{j}}$, then we accept the hypothesis of conditional independence between $Y^{i}$ and ${\langle Y^{ij}\rangle }_{j}$; otherwise, we reject it. We express this idea as the *TEA* score:

(28) $$g_{\mathrm{TEA}}\left(B:D\right)=\sum_{i=1}^{M}\left(2NT_{{\left\{Y^{ij}\right\}}_{j}\to Y^{i}}-\chi_{\alpha ,{\left\{l^{ij}\right\}}_{j}}\right).$$

In general, we only have access to *continuous* measurements of dynamical systems, and so are limited by the discrete or linear-Gaussian assumption. We can, however, use *surrogate* measurements $T_{{\langle Y^{ij}\rangle }_{j}^{s}\to Y^{i}}$ to empirically compute the distribution under the assumption of $H_{0}$ [52]. This same technique has been used by [38] to derive a greedy structure learning algorithm for effective network analysis. Here, ${\langle Y^{ij}\rangle }_{j}^{s}$ are surrogate sets of variables for ${\langle Y^{ij}\rangle }_{j}$, which have the same statistical properties as ${\langle Y^{ij}\rangle }_{j}$, but the correlation between ${\langle Y^{ij}\rangle }_{j}^{s}$ and $Y^{i}$ is removed. Let the distribution of these surrogate measurements be represented by some general function $T(s^{i})$ where, for the discrete and linear-Gaussian systems, we could compute $T(s^{i})$ analytically as an independent set of $\chi^{2}$-distributions $\chi^{2}\left({\left\{l^{ij}\right\}}_{j}\right)$. When no analytic distribution is known, we use a resampling method (i.e., permutation or bootstrapping), creating a large number of surrogate time-series pairs $\left\{{\langle Y^{ij}\rangle }_{j}^{s},Y^{i}\right\}$ by shuffling (for permutations, or redrawing for bootstrapping) the samples of $Y^{i}$ and computing a population of $T_{{\langle Y^{ij}\rangle }_{j}^{s}\to Y^{i}}$. As with the *TEA* score, we fix some confidence level α and determine the value $T_{\alpha ,s^{i}}$, such that $p(T\left(s^{i}\right)\leq T_{\alpha ,s^{i}})=\alpha$. This results in the *TEE* scoring function as

(29) $$g_{\mathrm{TEE}}\left(B:D\right)=\sum_{i=1}^{M}\left(T_{{\left\{Y^{ij}\right\}}_{j}\to Y^{i}}-T_{\alpha ,s^{i}}\right).$$

We can obtain the value $T_{\alpha ,s^{i}}$ by (1) drawing *S* samples $T_{{\langle Y^{ij}\rangle }_{j}^{s}\to Y^{i}}$ from the distribution $T(s^{i})$ (by permutation or bootstrapping), (2) fixing α∈{0,1/S,2/S,…,1}, and then (3) taking $T_{\alpha ,s^{i}}$ such that

$$\alpha =\frac{1}{S}\sum_{T_{{\left\{Y^{ij}\right\}}_{j}\to Y^{i}}}1_{T_{{\left\{Y^{ij}\right\}}_{j}^{s}\to Y^{i}}\leq T_{\alpha ,s^{i}}}.$$

We can alternatively limit the number of surrogates *S* to ⌈α/(1−α)⌉ and take the maximum as $T_{\alpha ,s^{i}}$ [22]; however, taking a larger number of surrogates will improve the validity of the distribution $T(s^{i})$.

**Figure 3:**
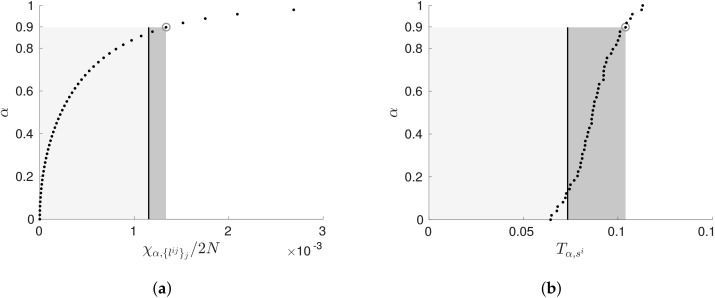
Distributions of the (**a**) *TEA* penalty function (28) and the (**b**) *TEE* penalty function (28). Both distributions were generated by observing the outcome of 1000 samples from two Gaussian variables with a correlation of 0.05. The figures illustrate: the distribution as a set of 100 sampled points (black dots); the area considered independent (grey regions); the measured transfer entropy (black line); and the difference between measurement and penalty term (dark grey region). Both tests use a value of α=0.9 (a *p*-value of 0.1). The distribution in (**a**) was estimated by assuming variables were linearly-coupled Gaussians, and the distribution in (**b**) was computed via a kernal box method (computed by the Java Information Dynamics Toolkit (JIDT), see [52] for details).

Both the analytical (*TEA*) and empirical (*TEE*) scoring functions are illustrated in Figure 3. Note that the approach of significance testing is functionally equivalent to considering the log-likelihood ratio in (6), where, as stated, nested log-likelihoods (and thus transfer entropy) follows the above $\chi^{2}$-distribution [48].

### 5.2. Implementation Details and Algorithm Analysis

The two main implementation challenges that arise when performing structure learning are: (1) computing the score for every candidate network and (2) obtaining a sufficient number of samples to recover the network. The main contributions of this work are theoretical justifications for measures already in use and, fortunately, algorithmic performance has already been addressed extensively using various heuristics. Here, we present an exact, exhaustive implementation for the purpose of validating our theoretical contributions.

First, for computing collective transfer entropy for the score (29), we require CPDs to be estimated from data. Given these CPDs, collective transfer entropy (A8) decomposes as a sum of *p* conditional transfer entropy (A7) terms, where $p=|{\left\{Y^{ij}\right\}}_{j}|$ is the size of the parent set (see Appendix B for details). Since most observations of dynamical systems are expected to be continuous, we employ a non-parametric, nearest-neighbour based approach to density estimation called the Kraskov–Stögbauer–Grassberger (KSG) estimator [43]. For any arbitrary decomposition of collective transfer entropy (i.e., any ordering of the parent set), this density estimation can be computed in time $O(\kappa \left(p+1\right)KN^{\kappa (p+1)}\log\left(N\right))$, where *K* is the number of nearest neighbours for each observation in a dataset of size *N*, and κ is the embedding dimension [52]. We upper bound this as $O(\kappa MKN^{\kappa M}\log\left(N\right))$ since the maximum *p* is M−1.

Now, the above density estimation was described for an arbitrary ordering of the parent set. In the case of parametric (discrete or linear-Gaussian) density estimation, every permutation of the parent set yields equivalent results, with potentially different $\chi_{\alpha ,{\left\{l^{ij}\right\}}_{j}}$ values for each permutation [3]; however, this is not the case for non-parametric density estimation techniques, e.g., the KSG estimator. Hence, as a conservative estimate of the score, we compute all p! permutations of the parent set and take the minimum collective transfer entropy. In order to obtain the surrogate distribution, we require *S* uncorrelated samples of the density. Since the surrogate distributions decompose in a similar manner, the score for a candidate network can be computed in time $O(S\cdot M!\cdot \kappa MKN^{\kappa M}\log\left(N\right))$, where, again, we have upper bounded p! as M!.

Using this approach, we can now compute the score (29), and thus the optimal graph $G^{*}$ can be found using any search procedure over DAGs. Exhaustive search, where all DAGs are enumerated, is typically intractable because the search space is super-exponential in the number of variables (about $2^{O(M^{2})}$), and so heuristics are often applied for efficiency. We restrict our attention to a relatively small network (a maximum of M=4 nodes) and thus we are able employ the dynamic programming (DP) approach of Silander and Myllymaki [53] to search through the space of all DAGs efficiently. This approach requires first computing the scores for all local parent sets, i.e., $2^{M}$ scores. Once each score is calculated, the DP algorithm runs in time $o(M\cdot 2^{M-1})$ and the entire search procedure run in time $O(M\cdot 2^{M-1}+2^{M}\cdot S\cdot M!\cdot \kappa MKN^{\kappa M}\log\left(N\right))$. As a consequence, the time complexity of the exhaustive algorithm is dominated by computing the $2^{M}$ scores and, in smaller networks, most of the time is spent on density estimation for surrogate distributions.

Finally, the problem of inferring optimal embedding parameters is well studied in the literature. In our experimental evaluation, we set the embedding dimension to the maximum, i.e., κ=2d+1, where *d* is the dimensionality of the entire latent state space (e.g., if M=3 and $d^{i}=3$ for each subsystem, then $\kappa =2\sum_{i}d^{i}+1=19$). However, determining these parameters would give more insight into the system and reduce the number of samples required for inference. There are numerous criteria for optimising these parameters (e.g., [54]); most notably, the work of [55] suggests an information-theoretic approach that could be integrated into the scoring function (29) to search over the embedding parameters and DAG space simultaneously.

## 6. Experimental Validation

The dynamics (1) and observation (2) maps can be obtained by either differential equations, discrete-time maps, or real-world measurements. To validate our approach, we use the toy example of distributed flows, whereby the dynamics of each node are given by either the Lorenz [56] or the Rössler system of ODEs [57]. The discrete-time measurements are obtained by integrating these ODEs over constant intervals. In this section, we formally introduce this model, study the effect of changing the parameters of a coupled Lorenz–Rössler system, and finally apply our scoring function to learn the structure of up to four coupled Lorenz attractors with arbitrary graph topology. To compute the scores, we use the Java Information Dynamics Toolkit (JIDT) [52], which includes both the KSG estimator and methods for generating the surrogate distributions.

### 6.1. Distributed Lorenz and Rössler Attractors

For validating our scoring function, we study coupled Lorenz and Rössler attractors. The Lorenz attractor exhibits chaotic solutions for certain parameter values and has been used to describe numerous phenomena of practical interest [56,58,59]. Each Lorenz system comprises three components ($d^{i}=3$), which we denote x=〈u,v,w〉; the state dynamics are given by:

(30) $$\dot{x}=g\left(x\right)=\left\{\begin{array}{c} \dot{u}=\sigma \left(v-u\right),\\ \dot{v}=u\left(\rho -w\right)-v,\\ \dot{w}=uv-\beta w, \end{array}\right.\;$$

with free parameters {σ,ρ,β}. Similarly, the Rössler attractor has state dynamics given by:

(31) $$\dot{x}=g\left(x\right)=\left\{\begin{array}{c} \dot{u}=-y-z,\\ \dot{v}=x+ay,\\ \dot{w}=b+z\left(x-c\right), \end{array}\right.\;$$

with free parameters {a,b,c} [57].

**Figure 4:**
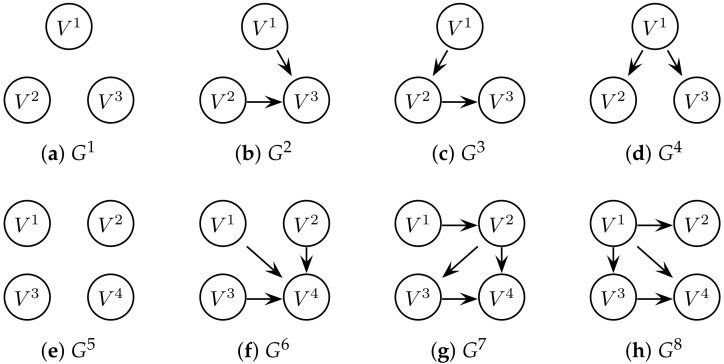
The network topologies used in this paper. The top row (**a**–**d**) are four arbitrary networks with three nodes (M=3) and the bottom row (**e**–**h**) are four arbitrary networks with four nodes (M=4).

In the distributed case, the components of each state vector $x_{t}^{i}$ are also driven by components of another subsystem. A number of different schemes have been proposed for coupling these variables, e.g., using the product [21,60] and the difference [61,62] of components. Our model uses the latter approach of linear differencing between one or more subsystem variables to couple the network. Let λ denote the coupling strength, *C* denote a three-dimensional vector of binary values, and *A* denote an adjacency (coupling) matrix (i.e., an M×M matrix of zeros with $A_{ij}=1$ iff $V^{i}\in \Pi_{G}\left(V^{j}\right)$). Then, the state equations for *M* spatially distributed systems can be expressed as

(32) $${\dot{x}}_{t}^{i}=g^{i}\left(x_{t}^{i}\right)+\nu_{f}+\lambda C\sum_{j=1}^{M}A_{ij}\left(x_{t}^{j}-x_{t}^{i}\right),\;$$

where $g^{i}\left(\cdot \right)$ represents the *i*-th chaotic attractor and $\nu_{f}$ is additive noise. In our simulations, we use λ=2, C=〈1,0,0〉 (each subsystem is coupled via variable *u*), and the adjacency matrices shown in Figure 4. In our experiments, we use common parameters for both attractors, i.e., σ=10,β=8/3,ρ=28 and a=0.1,b=0.1,c=14. For the observation $y_{t}^{i}$, it is common to use one component of the state as the read-out function [4,32,33]; we therefore let $y_{t}^{i}=u_{t}^{i}+\nu_{\psi }$. The noise terms are normally distributed with $\nu_{f}∼N\left(0,\sigma_{f}\right)$ and $\nu_{\psi }∼N\left(0,\sigma_{\psi }\right)$. Figure 1 illustrates example trajectories of Lorenz–Lorenz attractors coupled via this model.

### 6.2. Case Study: Coupled Lorenz–Rössler System

In order to characterise the effect of coupling on our score, we begin our evaluation by measuring the transfer entropy of a coupled Lorenz–Rössler attractor. In this setup, M=2, $\Pi_{G}\left(V^{1}\right)=\emptyset$, and $\Pi_{G}\left(V^{2}\right)=V^{1}$, $g^{1}\left(x\right)$ was given by (30), and $g^{2}\left(x\right)$ was given by (31). The transfer entropy was computed with a finite sample size of N=100,000.

**Figure 5:**
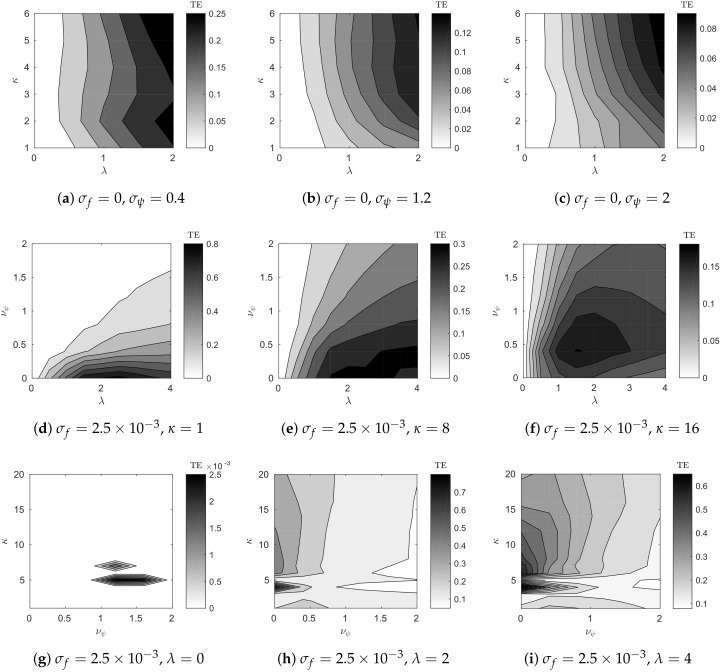
Transfer entropy as a function of the parameters of a coupled Lorenz–Rössler system. These components are: coupling strength λ and embedding dimension κ in the top row (**a**–**c**); coupling strength λ and observation noise $\sigma_{\psi }$ in the middle row (**d**–**f**); and observation noise $\sigma_{\psi }$ and embedding dimension κ in the bottom row (**g**–**i**).

Figure 5 shows the transfer entropy as a function of numerous parameters. In particular, the figure illustrates the effect of varying the coupling strength λ, embedding dimension κ, dynamics noise $\sigma_{f}$, and observation noise $\sigma_{\psi }$. As expected, increasing λ, or reducing either noise σ, increases the transfer entropy. The embedding dimension, however, increases to a set point, remains approximately constant, and then decreases. The κ-value above which transfer entropy remains constant illustrates the embedding dimension at which the dynamics are reconstructed; the decrease in transfer entropy after this point, however, is likely due to the finite sample size used for density estimation.

There are two interesting features in Figure 5 due to the dynamical systems studied. First, in the bottom row (Figure 5g–i), there is a bifurcation around κ=6. The theoretical embedding dimension for this system is $\kappa =2(d^{1}+d^{2})+1=7$, and, in this case, for κ<6, the embedding does not suffice to reconstruct the dynamics. Second, in Figure 5i, the transfer entropy decreases after about λ=2. This appears to be the case of synchrony due to strong coupling, where the dynamics of the forced variable become subordinate to the forcing [4], thus reducing the information transferred between the two subsystems.

### 6.3. Case Study: Network of Lorenz Attractors

In this section, we evaluate the score (27) in learning the structure of distributed dynamical systems. We will look at systems of three and four nodes of coupled Lorenz subsystems with arbitrary topologies. Unfortunately, significantly higher number of nodes become computationally expensive due to an increased embedding dimension κ, number of data points *N*, and number of permutations required to calculate the collective transfer entropy. To evaluate the performance of the score (27), the dynamics noise is constant $\sigma_{f}=0.01$, whereas the observation noise $\sigma_{\psi }$ and the number of observations taken *N* are varied. We selected the theoretical maximum embedding dimension κ=2d+1 and τ=1 as is common given discrete-time measurements [22]. It should be noted that from the results from Section 6.2 that transfer entropy is sensitive to the numerous parameters used to generate the data, and thus depending on the scenario, a significant sample size can be required for recovering the underlying graph structure. We do not make an effort to reduce this sample size and instead show the effect of using a different number of samples on the accuracy of the structure learning procedure.

In order to evaluate the scoring function, we compute the recall (R, or true positive rate), fallout (F, or false positive rate), and precision (P, or positive predictive value) of the recovered graph. Let TP denote the number of true positives (correct edges); TN denote the number of true negatives (correctly rejected edges); FP denote the number of false positives (incorrect edges); and FN denote the number of false negatives (incorrectly rejected edges). Then, R=TP/(TP+FN), F=FP/(FP+TN), and P=TP/(TP+FP). Finally, the $F_{1}$-score gives the harmonic mean of precision and recall to give a measure of the tests accuracy, i.e., $F_{1}=2\cdot R\cdot P/\left(R+P\right)$. Note that the ideal recall, precision and $F_{1}$-score is 1, and ideal fallout is 0. Furthermore, a ratio of R/F >1 suggests the classifier is better than random. As a summary statistic, Table 1 and Table 2 presents the $F_{1}$-scores for all networks illustrated in Figure 4, and the full classification results (e.g., precision, recall, and fallout) are given in Appendix C. The $F_{1}$-scores are thus a measure of how relevant the recovered network is to the original (generating) network from our data-driven approach.

In general, the results of Table 1 and Table 2 show that the scoring function is capable of recovering the network with high precision and recall, as well as low fallout. In the table, the cell colours are shaded to indicate higher (white) to lower (black) $F_{1}$ scores. The best performing score is that with a *p*-value of 0.01 and no penalisation (a *p*-value of *∞*) has the second highest classification results. As expected, the graphs recovered from data with low observational noise ($\sigma_{\psi }=1$) are more accurate than those inferred from noisier data ($\sigma_{\psi }=10$). The results for three-node networks (shown in Table 1) yields mostly full recovery of the structure for a higher number of observations N≥ 75 K, whereas, the four-node networks (shown in Table 2) are more difficult to classify.

Interestingly, the statistical significance testing does not have a strong effect on the results. It is unclear if this is due to the use of the non-parametric density estimators, which, in effect, are parsimonious in nature since transfer entropy will likely reduce when conditioning on more variables with a fixed samples size. One challenging case is the empty networks $G^{1}$ and $G^{5}$; this is shown in Appendix C, where the fallout is rarely 0 for any of the *p*-values or sample sizes (although a large number of observations N=100 K appears to reduce spurious edges). It would be expected that significance testing on these networks would outperform the naive score (27) given that a non-zero bias is introduced for a finite number of observations. Further investigation is required to understand why the null case fails.

## 7. Discussion and Future Work

We have presented a principled method to compute the KL divergence for model selection in distributed dynamical systems based on concepts from differential topology. The results presented in Figure 5 and Table 1 and Table 2 illustrate that this approach is suitable for recovering synchronous GDSs from data. Further, KL divergence is related to model encoding, which is a fundamental measure used in complex systems analysis. Our result, therefore, has potential implications for other areas of research. For example, the notion of equivalence classes in BN structure learning [63] should lend insight into the area of effective network analysis [35,36].

More specifically, the approach proposed here complements explicit Bayesian identification and comparison of state space models. In DCM, and more generally in approximate Bayesian inference, models are identified in terms of their parameters via an optimisation of an approximate posterior density over model parameters with respect to a variational (free energy) bound on log evidence [64]. After these parameters have been identified, this bound can be used directly for model comparison and selection. Interestingly, free energy is derived from the KL divergence between the approximate and true posterior and thus automatically penalises more complex models; however, in Equation (8), these distributions are inverted. In future work, it would be interesting to explore the relationship between transfer entropy and the variational free energy bound. Specifically, computing an evidence bound directly from the transfer entropy may allow us to avoid the significance testing described in Section 5 and instead use an approximation to evidence for structure learning.

Multivariate extensions to transfer entropy are known to eliminate redundant pairwise relationships and take into account the influence of confounding relationships in a network (i.e., synergistic effects) [65,66]. In this work, we have shown that this intuition holds for distributed dynamical systems when confined to a DAG topology. We conjecture that these methods are also applicable when cyclic dependencies exist within a graph, given any generic observation can be used in reconstructing the dynamics [50]; however, the methods presented are more likely to reveal *one* source in the cycle, rather than all information sources due to redundancy.

There are a number of extensions that should be considered for further practical implementations of this algorithm. Currently, we assume that the dimensionality of each subsystem is known, and thus we can bound the embedding dimension κ for recovering the hidden structure. However, this is generally infeasible in practice and a more general algorithm would infer the embedding dimension and time delay for an unknown system. Fortunately, there are numerous techniques to recover these parameters [54,55]. Furthermore, evaluating the quality of large graphs is infeasible with our current approach. However, our exact algorithm illustrates the feasibility of state space reconstruction in recovering a graph in practice. In the future, we aim to leverage the structure learning literature on reducing the search space and approximating scoring functions to produce more efficient algorithms.

Finally, the theoretical results of this work supplements understanding in fields where transfer entropy is commonly employed. Point processes are being increasingly viewed as models for a variety of information processing systems, e.g., as spiking neural trains [67] and adversaries in robotic patrolling models [68]. It was recently shown how transfer entropy can be computed for continuous time point processes such as these [67], allowing for efficient use of our analytical scoring function $g_{\mathrm{TEA}}$ in a number of contexts. Another intriguing line of research is the physical and thermodynamic interpretation of transfer entropy [69], particularly its relationship to the arrow of time [70]; this relationship between endomorphisms as discussed here and time asymmetry of thermodynamics should be explored further.

## Figures and Tables

**Table 1:** $F_{1}$-scores for three-node (M=3) networks. We present the classification summary for the three arbitrary topologies of coupled Lorenz systems represented by Figure 4b–d (network $G^{1}$ has no edges and thus an undefined $F_{1}$-score). The *p*-value of the *TEE* score is given in the top row of each table, with *∞* signifying using no significance testing, i.e., score (27).

		p=∞		p=0.01		p=0.001		p=0.0001	
Graph	*N*	$\sigma_{\psi }=1$	$\sigma_{\psi }=10$	$\sigma_{\psi }=1$	$\sigma_{\psi }=10$	$\sigma_{\psi }=1$	$\sigma_{\psi }=10$	$\sigma_{\psi }=1$	$\sigma_{\psi }=10$
$G^{2}$	5 K	0.8	0.5	0.8	0.5	0.8	0.5	0.8	0.5
	25 K	1	0.8	1	0.5	1	0.5	1	0.8
	100 K	1	0.5	1	1	1	1	1	0.8
$G^{3}$	5 K	1	0.67	1	1	1	1	1	0.67
	25 K	1	1	1	0.5	1	1	1	1
	100 K	1	1	1	1	1	1	1	1
$G^{4}$	5 K	0.8	-	0.8	0.8	0.8	0.5	0.8	-
	25 K	1	1	1	1	1	0.5	1	1
	100 K	1	1	1	1	1	1	1	1

**Table 2:** $F_{1}$-scores for four-node (M=4) networks. We present the classification summary for the three arbitrary topologies of coupled Lorenz systems represented by Figure 4f–h (network $G^{5}$ has no edges and thus an undefined $F_{1}$-score). The *p*-value of the *TEE* score is given in the top row of each table, with *∞* signifying using no significance testing, i.e., score (27).

		p=∞		p=0.01		p=0.001		p=0.0001	
Graph	*N*	$\sigma_{\psi }=1$	$\sigma_{\psi }=10$	$\sigma_{\psi }=1$	$\sigma_{\psi }=10$	$\sigma_{\psi }=1$	$\sigma_{\psi }=10$	$\sigma_{\psi }=1$	$\sigma_{\psi }=10$
$G^{6}$	5 K	0.57	0.5	0.57	0.29	0.57	0.29	0.57	-
	25 K	0.75	0.33	0.75	0.33	0.75	0.29	0.75	0.33
	100 K	1	0.33	1	0.57	1	0.4	1	0.33
$G^{7}$	5 K	1	0.25	1	0.29	0.75	0.25	0.75	0.57
	25 K	1	0.5	1	0.86	1	0.86	1	0.5
	100 K	1	0.86	1	0.86	1	0.86	1	0.86
$G^{8}$	5 K	1	0.25	1	0.57	1	0.75	1	0.25
	25 K	1	0.86	1	0.86	1	0.86	1	0.86
	100 K	1	0.86	1	0.86	1	0.57	1	0.86

## Appendix A. Embedding Theory

We refer here to embedding theory as the study of inferring the (hidden) state $x_{n}\in M$ of a dynamical system from a sequence of observations $y_{n}\in R$. This section will cover reconstruction theorems that define the conditions under which we can use delay embeddings for recovering the original dynamics *f* from this observed time series.

In differential topology, an *embedding* refers to a smooth map Φ:M→N between manifolds M and N if it maps M diffeomorphically onto its image. In Takens’ seminal work on turbulent flow [31], he proposed a map $\Phi_{f,\psi }:M\to R^{\kappa }$, that is composed of delayed observations, can be used to reconstruct the dynamics for typical (f,ψ). That is, fix some κ (the *embedding dimension*) and τ (the *time delay*), the *delay embedding map*, given by

(A1) $$\Phi_{f,\psi }\left(x_{n}\right)=y_{n}^{\left(\kappa \right)}=\langle y_{n},y_{n+\tau },y_{n+2\tau },\ldots ,y_{n+(\kappa -1)\tau }\rangle ,$$

is an embedding. More formally, denote $\Phi_{f,\psi }$, $D^{r}\left(M,M\right)$ as the space of $C^{r}$-diffeomorphisms on M and $C^{r}\left(M,R\right)$ as the space of $C^{r}$-functions on M, then the theorem can be expressed as follows.

**Theorem** **A1** (Delay Embedding Theorem for Diffeomorphisms [31]). Let M be a compact manifold of dimension d≥1. If κ≥2d+1 and r≥1, then there exists an open and dense set $\left(f,\psi \right)\in D^{r}\left(M,M\right)\times C^{r}\left(M,R\right)$ for which the map $\Phi_{f,\psi }$ is an embedding of M into $R^{\kappa }$.

The implication of Theorem 1 is that, for typical (f,ψ), the image $\Phi_{f,\psi }\left(M\right)$ of M under the delay embedding map $\Phi_{f,\psi }$ is completely equivalent to M itself, apart from the smooth invertible change of coordinates given by the mapping $\Phi_{f,\psi }$. An important consequence of this result is that we can define a map $F=\Phi_{f,\psi }\circ f\circ \Phi_{f,\psi }^{-1}$ on $\Phi_{f,\psi }$, such that $y_{n+1}^{\left(\kappa \right)}=F\left(y_{n}^{\left(\kappa \right)}\right)$ [44]. The bound for the open and dense set referred to in Theorem A1 is given by a number of technical assumptions. Denote ${\left(Df\right)}_{x}$ as the derivative of function *f* at a point x in the domain of *f*. The set of periodic points *A* of *f* with period less than τ has finitely many points. In addition, the eigenvalues of ${\left(Df\right)}_{x}$ at each x in a compact neighbourhood *A* are distinct and not equal to 1.

Theorem A1 was established for diffeomorphisms $D^{r}$; by definition, the dynamics are thus invertible in time. Thus, the time delay τ in (A1) can be either positive (delay lags) or negative (delay leads). Takens later proved a similar result for endomorphisms, i.e., non-invertible maps that restricts the time delay to a negative integer. Denote by E(M,M) the set of the space of $C^{r}$-endomorphisms on M, then the reconstruction theorem for endomorphisms can be expressed as the following.

**Theorem** **A2** (Delay Embedding Theorem for Endomorphisms [71]). Let M be a compact m dimensional manifold. If κ≥2d+1 and r≥1, then there exists an open and dense set $\left(f,\psi \right)\in D^{r}\left(M,M\right)\times C^{r}\left(M,R\right)$ for which there is a map $\pi_{\kappa }:X_{\kappa }\to M$ with $\pi_{\kappa }\Phi_{f,\psi }=f^{\kappa -1}$. Moreover, the map $\pi_{\kappa }$ has bounded expansion or is Lipschitz continuous.

As a result of Theorem A2, a sequence of κ successive measurements from a system determines the system state *at the end* of the sequence of measurements [71]. That is, there exists an endomorphism $F=\Phi_{f,\psi }\circ f\circ \Phi_{f,\psi }^{-1}$ to predict the next observation if one takes a negative time (lead) delay τ in (A1).

In this work, we consider two important generalisations of the Delay Embedding Theorem A1. Both of these theorems follow similar proofs to the original and have thus been derived for diffeomorphisms, not endomorphisms. However, encouraging empirical results in [6] support the conjecture that they can both be generalised to the case of endomorphisms by taking a negative time delay, as is done in Theorem A2 above. This would allow for not only distributed flows that are used in our work, but endomorphic maps, e.g., the well-studied coupled map lattice structure [51].

The first generalisation is by Stark et al. [44] and deals with a skew-product system. That is, *f* is now forced by some second, independent system g:N→N. The dynamical system on M×N is thus given by the set of equations

(A2) $$x_{n+1}=f\left(x_{n},\omega_{n}\right),\;\omega_{n+1}=g\left(\omega_{n}\right).$$

In this case, the delay map is written as

(A3) $$\Phi_{f,g,\psi }\left(x,\omega \right)=\langle y_{n},y_{n+\tau },y_{n+2\tau },\ldots ,y_{n+(\kappa -1)\tau }\rangle ,$$

and the theorem can be expressed as follows.

**Theorem** **A3** (Bundle Delay Embedding Theorem [44]). Let M and N be compact manifolds of dimension d≥1 and e, respectively. Suppose that κ≥2(d+e)+1 and the periodic orbits of period ≤d of $g\in D^{r}\left(N\right)$ are isolated and have distinct eigenvalues. Then, for r≥1, there exists an open and dense set of $\left(f,\psi \right)\subset D^{r}\left(M\times N,M\right)\times C^{r}\left(M,R\right)$ for which the map $\Phi_{f,g,\psi }$ is an embedding of M×N into $R^{\kappa }$.

Finally, all theorems up until now have assumed a single read-out function for the system in question. Recently, Sugihara et al. [4] showed that multivariate mappings also form an embedding, with minor changes to the technical assumptions underlying Takens’ original theorem. That is, given M≤2d+1 different observation functions, the delay map can be written as

(A4) $$\Phi_{f,\langle \psi^{i}\rangle }\left(x\right)=\langle \Phi_{f,\psi^{1}}\left(x\right),\Phi_{f,\psi^{2}}\left(x\right),\ldots ,\Phi_{f,\psi^{M}}\left(x\right)\rangle ,$$

where each delay map $\Phi_{f,\psi^{i}}$ is as per (A1) for individual embedding dimension $\kappa^{i}\leq \kappa$. The theorem can then be stated as follows.

**Theorem** **A4** (Delay Embedding Theorem for Multivariate Observation Functions [50]). Let M be a compact manifold of dimension d≥1. Consider a diffeomorphism $f\in D^{r}\left(M,M\right)$ and a set of at most 2d+1 observation functions $\langle \psi^{i}\rangle$ where each $\psi^{i}\in C^{r}\left(M,R\right)$ and r≥2. If $\sum_{i}\kappa^{i}\geq 2d+1$, then, for generic $\left(f,\langle \psi^{i}\rangle \right)$, the map $\Phi_{f,\langle \psi^{i}\rangle }$ is an embedding.

## Appendix B. Information Theory

In this section, we introduce some key concepts of information theory: conditional entropy; conditional and collective transfer entropy; and stochastic interaction.

Consider two arbitrary random variables *X* and *Y*; the *conditional entropy* H(X∣Y) represents the uncertainty of *X* after taking into account the outcomes of another random variable *Y* by the equation

(A5) $$H\left(X|Y\right)=-\sum_{x,y}\mathrm{Pr}\left(x,y\right)\log\mathrm{Pr}\left(x|y\right)=E\left[\mathrm{Pr}(x|y)\right].$$

Transfer entropy detects the directed exchange of information between random processes by marginalising out common history and static correlations between variables; it is thus considered a measure of information transfer within a system [25]. Let the processes *X* and *Y* have associated embedding dimensions $\kappa^{X}$ and $\kappa^{Y}$. The *transfer entropy* of *X* to *Y* is given in terms of conditional entropy:

(A6) $$T_{X\to Y}=H\left(Y_{n+1}|Y_{n}^{\left(\kappa^{Y}\right)}\right)-H\left(Y_{n+1}|X_{n}^{i,(\kappa^{X})},Y_{n}^{\left(\kappa^{Y}\right)}\right).$$

Now, given a third process *Z* with embedding dimension $\kappa^{Z}$, we can compute the information transfer of *X* to *Y* in the context of *Z* as:

(A7) $$T_{X\to Y|Z}=H\left(Y_{n+1}|Y_{n}^{\left(\kappa^{Y}\right)},Z_{n}^{i,(\kappa^{Z})}\right)-H\left(Y_{n+1}|X_{n}^{i,(\kappa^{X})},Y_{n}^{\left(\kappa^{Y}\right)},Z_{n}^{i,(\kappa^{Z})}\right).$$

The collective transfer entropy computes the information transfer between a set of *M* source processes and a single destination process [19]. Consider the set $Y=\{Y^{i}\}$ of source processes. We can compute the collective transfer entropy from Y to the destination process *X* as a function of conditional entropy (A5) terms:

(A8) $$\begin{array}{cc} T_{Y\to X} & =T_{Y^{1}\to X}+\sum_{i=1}^{M}T_{Y^{i}\to X|\left\{Y^{1},\ldots ,Y^{i-1}\right\}}, \end{array}$$

where the ordering of the source processes are arbitrary.

Stochastic interaction measures the complexity of dynamical systems by quantifying the excess of information processed, in time, by the system beyond the information processed by each of the nodes [17,18,72,73]. Using the same notation, stochastic interaction of the collection of processes Y is

(A9) $$\begin{array}{cc} S_{Y} & =-H\left(Y_{n+1}|\left\{Y_{n}^{i,(\kappa^{i})}\right\}\right)+\sum_{i=1}^{M}H\left(Y_{n+1}^{i}|Y_{n}^{i,(\kappa^{i})}\right). \end{array}$$

The standard definition assumes a first-order Markov process [17,18]; In (A9), we generalise stochastic interaction to arbitrary κ-order Markov chains.

## Appendix C. Extended Results

Here, we present the extended results of Table 1 and Table 2. That is, we give the precision, recall, fallout, and $F_{1}$-scores for the eight networks of Lorenz attractors shown in Figure 4. These results are given for a number of different sample sizes to illustrate the sample complexity of this problem: N=5000 (Table A1 and Table A2), N= 10,000 (Table A3 and Table A4), N= 25,000 (Table A5 and Table A6), N= 50,000 (Table A7 and Table A8), and N= 100,000 (Table A9 and Table A10). Each table has results for various *p*-values (with a *p*-value of *∞* denoting the maximum likelihood score (27)), as well as two different observation noise variances, $\sigma_{\psi }=1$ and $\sigma_{\psi }=10$.

**Table A1:** Classification results for three-node (M=3) networks for N=5000 samples. We present the precision (P), recall (R), fallout (F), and $F_{1}$-score for the eight arbitrary topologies of coupled Lorenz systems represented by Figure 4.

Graph	*p*-Value	*∞*		0.01		0.001		0.0001	
	$\sigma_{\psi }$	1	10	1	10	1	10	1	10
$G^{1}$	*R*	-	-	-	-	-	-	-	-
	*F*	0.33	0.22	0.33	0.22	0.22	0.33	0.33	0.22
	*P*	0	0	0	0	0	0	0	0
	$F_{1}$	-	-	-	-	-	-	-	-
$G^{2}$	*R*	1	0.5	1	0.5	1	0.5	1	0.5
	*F*	0.14	0.14	0.14	0.14	0.14	0.14	0.14	0.14
	*P*	0.67	0.5	0.67	0.5	0.67	0.5	0.67	0.5
	$F_{1}$	0.8	0.5	0.8	0.5	0.8	0.5	0.8	0.5
$G^{3}$	*R*	1	0.5	1	1	1	1	1	0.5
	*F*	0	0	0	0	0	0	0	0
	*P*	1	1	1	1	1	1	1	1
	$F_{1}$	1	0.67	1	1	1	1	1	0.67
$G^{4}$	*R*	1	0	1	1	1	0.5	1	0
	*F*	0.14	0.43	0.14	0.14	0.14	0.14	0.14	0.43
	*P*	0.67	0	0.67	0.67	0.67	0.5	0.67	0
	$F_{1}$	0.8	-	0.8	0.8	0.8	0.5	0.8	-

**Table A2:** Classification results for four-node (M=4) networks for N=5000 samples. We present the precision (P), recall (R), fallout (F), and $F_{1}$-score for the eight arbitrary topologies of coupled Lorenz systems represented by Figure 4.

Graph	*p*-Value	*∞*		0.01		0.001		0.0001	
	$\sigma_{\psi }$	1	10	1	10	1	10	1	10
$G^{5}$	*R*	-	-	-	-	-	-	-	-
	*F*	0.31	0.25	0.31	0.19	0.31	0.25	0.31	0.19
	*P*	0	0	0	0	0	0	0	0
	$F_{1}$	-	-	-	-	-	-	-	-
$G^{6}$	*R*	0.67	0.67	0.67	0.33	0.67	0.33	0.67	0
	*F*	0.15	0.23	0.15	0.23	0.15	0.23	0.15	0.31
	*P*	0.5	0.4	0.5	0.25	0.5	0.25	0.5	0
	$F_{1}$	0.57	0.5	0.57	0.29	0.57	0.29	0.57	-
$G^{7}$	*R*	1	0.25	1	0.25	0.75	0.25	0.75	0.5
	*F*	0	0.25	0	0.17	0.083	0.25	0.083	0.083
	*P*	1	0.25	1	0.33	0.75	0.25	0.75	0.67
	$F_{1}$	1	0.25	1	0.29	0.75	0.25	0.75	0.57
$G^{8}$	*R*	1	0.25	1	0.5	1	0.75	1	0.25
	*F*	0	0.25	0	0.083	0	0.083	0	0.25
	*P*	1	0.25	1	0.67	1	0.75	1	0.25
	$F_{1}$	1	0.25	1	0.57	1	0.75	1	0.25

**Table A3:** Classification results for three-node (M=3) networks for N= 10,000 samples. We present the precision (P), recall (R), fallout (F), and $F_{1}$-score for the eight arbitrary topologies of coupled Lorenz systems represented by Figure 4.

Graph	*p*-Value	*∞*		0.01		0.001		0.0001	
	$\sigma_{\psi }$	1	10	1	10	1	10	1	10
$G^{1}$	*R*	-	-	-	-	-	-	-	-
	*F*	0.22	0.11	0.22	0.11	0.22	0.22	0.22	0.11
	*P*	0	0	0	0	0	0	0	0
	$F_{1}$	-	-	-	-	-	-	-	-
$G^{2}$	*R*	1	0.5	1	0.5	1	0.5	1	0.5
	*F*	0	0.14	0	0.14	0	0.14	0	0.14
	*P*	1	0.5	1	0.5	1	0.5	1	0.5
	$F_{1}$	1	0.5	1	0.5	1	0.5	1	0.5
$G^{3}$	*R*	1	0.5	1	1	1	0	1	0.5
	*F*	0	0.14	0	0	0	0.29	0	0.14
	*P*	1	0.5	1	1	1	0	1	0.5
	$F_{1}$	1	0.5	1	1	1	-	1	0.5
$G^{4}$	*R*	1	1	1	0.5	1	0.5	1	1
	*F*	0.14	0.14	0	0	0.14	0.14	0.14	0.14
	*P*	0.67	0.67	1	1	0.67	0.5	0.67	0.67
	$F_{1}$	0.8	0.8	1	0.67	0.8	0.5	0.8	0.8

**Table A4:** Classification results for four-node (M=4) networks for N= 10,000 samples. We present the precision (P), recall (R), fallout (F), and $F_{1}$-score for the eight arbitrary topologies of coupled Lorenz systems represented by Figure 4.

Graph	*p*-Value	*∞*		0.01		0.001		0.0001	
	$\sigma_{\psi }$	1	10	1	10	1	10	1	10
$G^{5}$	*R*	-	-	-	-	-	-	-	-
	*F*	0.31	0.25	0.31	0.19	0.31	0.19	0.31	0.25
	*P*	0	0	0	0	0	0	0	0
	$F_{1}$	-	-	-	-	-	-	-	-
$G^{6}$	*R*	0.67	0.33	0.67	0	1	1	0.67	0.33
	*F*	0.15	0.15	0.15	0.15	0.15	0.15	0.15	0.15
	*P*	0.5	0.33	0.5	0	0.6	0.6	0.5	0.33
	$F_{1}$	0.57	0.33	0.57	-	0.75	0.75	0.57	0.33
$G^{7}$	*R*	0.75	0.5	1	0.5	1	0.25	0.75	0.5
	*F*	0.083	0.083	0	0.083	0	0.17	0.083	0.083
	*P*	0.75	0.67	1	0.67	1	0.33	0.75	0.67
	$F_{1}$	0.75	0.57	1	0.57	1	0.29	0.75	0.57
$G^{8}$	*R*	1	0.25	1	0.25	1	0	1	0.25
	*F*	0	0.17	0	0.17	0	0.25	0	0.17
	*P*	1	0.33	1	0.33	1	0	1	0.33
	$F_{1}$	1	0.29	1	0.29	1	-	1	0.29

**Table A5:** Classification results for three-node (M=3) networks for N= 25,000 samples. We present the precision (P), recall (R), fallout (F), and $F_{1}$-score for the eight arbitrary topologies of coupled Lorenz systems represented by Figure 4.

Graph	*p*-Value	*∞*		0.01		0.001		0.0001	
	$\sigma_{\psi }$	1	10	1	10	1	10	1	10
$G^{1}$	*R*	-	-	-	-	-	-	-	-
	*F*	0.22	0.11	0.22	0.11	0.22	0.22	0.22	0.11
	*P*	0	0	0	0	0	0	0	0
	$F_{1}$	-	-	-	-	-	-	-	-
$G^{2}$	*R*	1	1	1	0.5	1	0.5	1	1
	*F*	0	0.14	0	0.14	0	0.14	0	0.14
	*P*	1	0.67	1	0.5	1	0.5	1	0.67
	$F_{1}$	1	0.8	1	0.5	1	0.5	1	0.8
$G^{3}$	*R*	1	1	1	0.5	1	1	1	1
	*F*	0	0	0	0.14	0	0	0	0
	*P*	1	1	1	0.5	1	1	1	1
	$F_{1}$	1	1	1	0.5	1	1	1	1
$G^{4}$	*R*	1	1	1	1	1	0.5	1	1
	*F*	0	0	0	0	0	0.14	0	0
	*P*	1	1	1	1	1	0.5	1	1
	$F_{1}$	1	1	1	1	1	0.5	1	1

**Table A6:** Classification results for four-node (M=4) networks for N= 25,000 samples. We present the precision (P), recall (R), fallout (F), and $F_{1}$-score for the eight arbitrary topologies of coupled Lorenz systems represented by Figure 4.

Graph	*p*-Value	*∞*		0.01		0.001		0.0001	
	$\sigma_{\psi }$	1	10	1	10	1	10	1	10
$G^{5}$	*R*	-	-	-	-	-	-	-	-
	*F*	0.31	0.19	0.31	0.19	0.31	0.19	0.31	0.19
	*P*	0	0	0	0	0	0	0	0
	$F_{1}$	-	-	-	-	-	-	-	-
$G^{6}$	*R*	1	0.33	1	0.33	1	0.33	1	0.33
	*F*	0.15	0.15	0.15	0.15	0.15	0.23	0.15	0.15
	*P*	0.6	0.33	0.6	0.33	0.6	0.25	0.6	0.33
	$F_{1}$	0.75	0.33	0.75	0.33	0.75	0.29	0.75	0.33
$G^{7}$	*R*	1	0.5	1	0.75	1	0.75	1	0.5
	*F*	0	0.17	0	0	0	0	0	0.17
	*P*	1	0.5	1	1	1	1	1	0.5
	$F_{1}$	1	0.5	1	0.86	1	0.86	1	0.5
$G^{8}$	*R*	1	0.75	1	0.75	1	0.75	1	0.75
	*F*	0	0	0	0	0	0	0	0
	*P*	1	1	1	1	1	1	1	1
	$F_{1}$	1	0.86	1	0.86	1	0.86	1	0.86

**Table A7:** Classification results for three-node (M=3) networks with N= 50,000 samples. We present the precision (P), recall (R), fallout (F), and $F_{1}$-score for the eight arbitrary topologies of coupled Lorenz systems represented by Figure 4.

Graph	*p*-Value	*∞*		0.01		0.001		0.0001	
	$\sigma_{\psi }$	1	10	1	10	1	10	1	10
$G^{1}$	*R*	-	-	-	-	-	-	-	-
	*F*	0	0.11	0	0	0	0.11	0	0.22
	*P*	-	0	-	-	-	0	-	0
	$F_{1}$	-	-	-	-	-	-	-	-
$G^{2}$	*R*	1	0.5	1	0.5	1	0.5	1	0.5
	*F*	0	0.14	0	0.14	0	0.14	0	0.14
	*P*	1	0.5	1	0.5	1	0.5	1	0.5
	$F_{1}$	1	0.5	1	0.5	1	0.5	1	0.5
$G^{3}$	*R*	1	1	1	0.5	1	1	1	1
	*F*	0	0.14	0	0.14	0	0.14	0	0
	*P*	1	0.67	1	0.5	1	0.67	1	1
	$F_{1}$	1	0.8	1	0.5	1	0.8	1	1
$G^{4}$	*R*	1	0.5	1	1	1	0.5	1	1
	*F*	0	0.14	0	0	0	0.14	0	0
	*P*	1	0.5	1	1	1	0.5	1	1
	$F_{1}$	1	0.5	1	1	1	0.5	1	1

**Table A8:** Classification results for four-node (M=4) networks with N= 50,000 samples. We present the precision (P), recall (R), fallout (F), and $F_{1}$-score for the eight arbitrary topologies of coupled Lorenz systems represented by Figure 4.

Graph	*p*-Value	*∞*		0.01		0.001		0.0001	
	$\sigma_{\psi }$	1	10	1	10	1	10	1	10
$G^{5}$	*R*	-	-	-	-	-	-	-	-
	*F*	0.19	0.062	0.19	0.19	0.19	0.12	0.19	0.12
	*P*	0	0	0	0	0	0	0	0
	$F_{1}$	-	-	-	-	-	-	-	-
$G^{6}$	*R*	1	0.33	1	0	1	0.33	1	0.33
	*F*	0	0.15	0	0	0	0.23	0.15	0.15
	*P*	1	0.33	1	-	1	0.25	0.6	0.33
	$F_{1}$	1	0.33	1	-	1	0.29	0.75	0.33
$G^{7}$	*R*	1	0.75	1	0.5	1	0.5	1	0.75
	*F*	0	0	0	0.17	0	0.083	0	0
	*P*	1	1	1	0.5	1	0.67	1	1
	$F_{1}$	1	0.86	1	0.5	1	0.57	1	0.86
$G^{8}$	*R*	1	0.75	1	0.75	1	0.75	1	0.75
	*F*	0	0	0	0	0	0	0	0
	*P*	1	1	1	1	1	1	1	1
	$F_{1}$	1	0.86	1	0.86	1	0.86	1	0.86

**Table A9:** Classification results for three-node (M=3) networks with N= 100,000 samples. We present the precision (P), recall (R), fallout (F), and $F_{1}$-score for the eight arbitrary topologies of coupled Lorenz systems represented by Figure 4.

Graph	*p*-Value	*∞*		0.01		0.001		0.0001	
	$\sigma_{\psi }$	1	10	1	10	1	10	1	10
$G^{1}$	*R*	-	-	-	-	-	-	-	-
	*F*	0	0.22	0	0.11	0	0.22	0	0.11
	*P*	-	0	-	0	-	0	-	0
	$F_{1}$	-	-	-	-	-	-	-	-
$G^{2}$	*R*	1	0.5	1	1	1	1	1	1
	*F*	0	0.14	0	0	0	0	0	0.14
	*P*	1	0.5	1	1	1	1	1	0.67
	$F_{1}$	1	0.5	1	1	1	1	1	0.8
$G^{3}$	*R*	1	1	1	1	1	1	1	1
	*F*	0	0	0	0	0	0	0	0
	*P*	1	1	1	1	1	1	1	1
	$F_{1}$	1	1	1	1	1	1	1	1
$G^{4}$	*R*	1	1	1	1	1	1	1	1
	*F*	0	0	0	0	0	0	0	0
	*P*	1	1	1	1	1	1	1	1
	$F_{1}$	1	1	1	1	1	1	1	1

**Table A10:** Classification results for four-node (M=4) networks with N= 100,000 samples. We present the precision (P), recall (R), fallout (F), and $F_{1}$-score for the eight arbitrary topologies of coupled Lorenz systems represented by Figure 4.

Graph	*p*-Value	*∞*		0.01		0.001		0.0001	
	$\sigma_{\psi }$	1	10	1	10	1	10	1	10
$G^{5}$	*R*	-	-	-	-	-	-	-	-
	*F*	0.19	0.062	0.19	0.062	0.19	0.19	0.19	0.12
	*P*	0	0	0	0	0	0	0	0
	$F_{1}$	-	-	-	-	-	-	-	-
$G^{6}$	*R*	1	0.33	1	0.67	1	0.33	1	0.33
	*F*	0	0.15	0	0.15	0	0.077	0	0.15
	*P*	1	0.33	1	0.5	1	0.5	1	0.33
	$F_{1}$	1	0.33	1	0.57	1	0.4	1	0.33
$G^{7}$	*R*	1	-	1	-	1	-	1	-
	*F*	0	-	0	-	0	-	0	-
	*P*	1	-	1	-	1	-	1	-
	$F_{1}$	1	-	1	-	1	-	1	-
$G^{8}$	*R*	1	0.75	1	0.75	1	0.5	1	0.75
	*F*	0	0	0	0	0	0.083	0	0
	*P*	1	1	1	1	1	0.67	1	1
	$F_{1}$	1	0.86	1	0.86	1	0.57	1	0.86
